# Enhanced immunogenicity and protective efficacy in mice following a Zika DNA vaccine designed by modulation of membrane-anchoring regions and its association to adjuvants

**DOI:** 10.3389/fimmu.2024.1307546

**Published:** 2024-01-19

**Authors:** Franciane Mouradian Emidio Teixeira, Luana de Mendonça Oliveira, Anna Cláudia Calvielli Castelo Branco, Ricardo Wesley Alberca, Emanuella Sarmento Alho de Sousa, Bruno Henrique de Sousa Leite, Wenny Camilla dos Santos Adan, Alberto José da Silva Duarte, Roberto Dias Lins, Maria Notomi Sato, Isabelle Freire Tabosa Viana

**Affiliations:** ^1^Laboratory of Dermatology and Immunodeficiencies, LIM-56, Department of Dermatology, Tropical Medicine Institute of São Paulo, University of São Paulo Medical School, São Paulo, Brazil; ^2^Department of Immunology, Institute of Biomedical Sciences, University of São Paulo, São Paulo, Brazil; ^3^Department of Virology, Aggeu Magalhães Institute, Oswaldo Cruz Foundation, Recife, Brazil

**Keywords:** DNA vaccine, Zika virus, envelope protein, membrane-anchoring regions, adjuvants, protection, immunogenicity

## Abstract

Zika virus (ZIKV) is a re-emerging pathogen with high morbidity associated to congenital infection. Despite the scientific advances since the last outbreak in the Americas, there are no approved specific treatment or vaccines. As the development of an effective prophylactic approach remains unaddressed, DNA vaccines surge as a powerful and attractive candidate due to the efficacy of sequence optimization in achieving strong immune response. In this study, we developed four DNA vaccine constructs encoding the ZIKV prM/M (pre-membrane/membrane) and E (envelope) proteins in conjunction with molecular adjuvants. The DNA vaccine candidate (called ZK_ΔSTP), where the entire membrane-anchoring regions were completely removed, was far more immunogenic compared to their counterparts. Furthermore, inclusion of the tPA-SP leader sequence led to high expression and secretion of the target vaccine antigens, therefore contributing to adequate B cell stimulation. The ZK_ΔSTP vaccine induced high cellular and humoral response in C57BL/6 adult mice, which included high neutralizing antibody titers and the generation of germinal center B cells. Administration of ZK-ΔSTP incorporating aluminum hydroxide (Alum) adjuvant led to sustained neutralizing response. In consistency with the high and long-term protective response, ZK_ΔSTP+Alum protected adult mice upon viral challenge. Collectively, the ZK_ΔSTP+Alum vaccine formulation advances the understanding of the requirements for a successful and protective vaccine against flaviviruses and is worthy of further translational studies.

## 1 Introduction

Zika virus (ZIKV) is a re-emerging member of the Flaviviridae family, which comprises other viruses of medical importance, such as dengue virus (DENV), West Nile virus, Japanese encephalitis virus (JEV) and yellow fever virus (YFV) (1). ZIKV infection still represents a serious public health concern. Although ZIKV infections are commonly mild or asymptomatic, severe neurological disorders have been observed in a number of cases. ZIKV targets neurological cells at different development stages from progenitor to mature cells (2). Although ZIKV is usually less neuroinvasive in adults (compared to fetus), the major neurological infection outcome in this population includes Guillain-Barré Syndrome (GBS) (3). Additionally, when the infection takes place during pregnancy, the viral tropism to neuronal progenitor and placenta cells leads to congenital Zika syndrome (CZS) in the developing fetus, which is characterized by microcephaly, structural brain abnormalities and cognitive impairment (4–7). Despite the progress in the development of vaccine candidates against ZIKV since the last outbreak in 2015, there are no licensed prophylactic vaccines. In addition, pregnant women represent the main risk group for ZIKV infection, as current literature shows evidence for ongoing virus circulation in the human population (8).

ZIKV genome codes for three structural proteins named capsid (C), pre-membrane/membrane (prM/M) and envelope (E). The latter is known as the most antigenic viral protein and is often considered as the best candidate for immunization and neutralizing antibodies (nAb) induction (9–11). Several early studies on ZIKV vaccines aimed to use the complete prM/M and E proteins to elicit optimal vaccine response and protection against adult infection (12–15). However, other studies have designed their formulations targeting specific E protein domains, which led to the identification of important regions that are more effective in stimulating a strong immune response (16, 17). Furthermore, the removal of E protein anchoring regions (e.g., the transmembrane (TM) portions) was shown to improve vaccine immunogenicity (18, 19).

DNA vaccines have evolved over time as a powerful therapeutic platform, accommodating qualities of high safety and stability and low production costs. Additionally, DNA vaccines allow for changes that can be easily performed to improve their efficacy, such as the incorporation of molecular adjuvants and delivery methods, and the optimization of signal sequences aimed towards better antigenic processing and presentation (20). Among the available molecular adjuvants, those which direct antigen presentation by MHC-II molecules (*e.g.*, the lysosomal associated membrane protein (LAMP)) as well as those that stimulate the secretion of folded proteins (e.g., tissue plasminogen activator signal peptide (tPA-SP) sequence) represent attractive choices for genetic vaccine formulations.

LAMP-based DNA vaccines have been broadly described for several viruses (18, 21–26), being capable of promoting strong antibody response. While LAMP-based DNA vaccines achieve high immunogenicity by promoting antigen processing and presentation through MHC-II, the proper B cell stimulation often requires the recognition of key epitopes that are only available in the context of the folded protein. For these cases, the tPA-SP leader sequence might be a more suitable choice since it promotes high level of protein expression and directs antigen secretion through the cellular secretion pathway (27). This approach has also been previously described to induce intense humoral and sustained cell-mediated response in DNA vaccine models for DENV (28, 29), influenza A (30), JEV (31), tuberculosis (32), HIV (33) and ZIKV (19).

In this work, we designed four DNA vaccine formulations coding for the prM/M and E proteins of ZIKV. Aiming to achieve strong and sustained immune response, antigen presentation was optimized through the addition of the LAMP and tPA molecular adjuvants. Additionally, the secretion of vaccine antigens in their native conformation (folded as in the viral particle) was achieved through the removal of the E protein membrane-anchoring regions. After initial *in vitro* and *in vivo* screenings, the immunogenicity and protective efficacy of best vaccine candidate was further experimentally validated in mouse model.

## 2 Materials and methods

### 2.1 Vaccine design

The amino acid sequence coding for the ZIKV prM/M and E proteins was retrieved from the NCBI (National Center for Biotechnology Information, accession number: KU321639.1) and four vaccine constructs comprising the full-length prM/M and E proteins were designed as follows: ZK_ΔTMP (comprising a deletion of the TM region); LAMP/ZK_ΔTMP (comprising a deletion of the TM region and the addition of the LAMP sequence); ZK_ΔSTP (comprising a deletion of the TM and stem regions) and LAMP/ZK_ΔSTP (comprising a deletion of the TM and stem regions and the addition of the LAMP sequence). The constructs were reverse translated to DNA and optimized for expression in eukaryotic cells. The expression cassettes were commercially synthesized and inserted into the pcDNA3.1 mammalian expression vector (Genscript, USA).

### 2.2 Detection of ZIKV E protein expression in cell culture by cell-based ELISA

Monolayers of BHK-21 cells (ATCC, USA) were transfected with the constructs using Lipofectamine 2000 (Invitrogen, USA) according to the manufacturer’s instructions. After 48h, cells were fixed using 80% acetone for 1h at room temperature (RT) and blocked with assay buffer containing 5% skimmed milk (Bio-Rad, USA) in PBS-T buffer (1X PBS with 0.05% Tween-20) for 1h at RT. For ZIKV E detection, the 4G2 monoclonal antibody (34) (1μg/mL) was added in assay buffer for 2h at 37°C. Plates were washed five times with PBS-T, followed by peroxidase-conjugated anti-mouse IgG antibody (Sigma Aldrich, USA) for 1h at RT. After a final wash step, the reaction was revealed with tetramethylbenzidine (TMB, KPL SureBlue Reserve, USA) substrate for 45 min at RT, stopped with 1N HCl and read at 450nm (OD 450nm) using a microplate spectrophotometer (Benchmark Plus, Bio-rad, USA). A plasmid coding for YFV proteins (pL/YFV) was used as a positive control (18). All samples were tested in duplicate, and the results were considered valid when intra-assay variability was below 20%.

### 2.3 Detection of soluble ZIKV E protein in cell culture supernatant by ELISA

The supernatant of transfected BHK21 cell culture was transferred to high binding, half area 96-well polystyrene plates (Costar, USA), and soluble ZIKV E proteins were allowed to bind to the plate surface for 16h at 4°C. Detection of immobilized ZIKV E proteins was performed following the ELISA protocol previously described.

### 2.4 Animals

Adult C57BL/6 mice (female and male), aged 8–10 weeks old, were purchased from the Faculdade de Medicina da Universidade de São Paulo (FMUSP, Brazil) animal facility and bred in specific pathogen-free conditions. Adult C57BL/6 mice with interferon α and β receptor depletion (IFNαβR^-/-^) (B6.129S2-Ifnar1tm1Agt/Mmjax- https://www.jax.org/strain/010830), known to be the susceptible model of ZIKV infection, were used for adult infection assays. The IFNαβR^-/-^ mice were purchased from Instituto de Ciências Biomédicas da Universidade de São Paulo (ICB-USP, Brazil). Protocols were approved by the local Ethics Committee of Animal Care and Use (CEUA-ICB: 6912040918, CEUA-IMT: 363 and CEUA-FMUSP: 1763/2022).

### 2.5 Mice immunization protocol

Adult mice received two doses of immunization by intradermal (id) route at a 20-days interval with 50 μg of the DNA plasmids in a total volume of 50 μL of PBS delivered at the base of the tail. PBS solution was used as negative control. For adjuvant-associated immunization, mice received two doses of 50 μg of ZK_ΔSTP vaccine in the presence of commercial adjuvant formulations, such as Alum (*i.e.*, aluminum hydroxide, 10 mg/mouse, Thermo Scientific, USA), MPLA (monophosphoryl lipid A, 10 μg/mouse, Sigma Aldrich, USA) or a combination of Alum (10 mg/mouse) + MPLA (10 μg/mouse). The solutions comprising the DNA vaccine along with each commercial adjuvants were freshly prepared immediately before immunization according to the manufacturers’ recommendation. Briefly, the plasmid DNA (50 μg) was diluted in each adjuvant suspension and gently homogenized by pipetting. Immunogenicity evaluation was performed at different time points after boost. Sera, inguinal lymph nodes (iLN) and spleens were also obtained at different time points.

### 2.6 ZIKV infection in adult mice

The Asian strain of locally isolated ZIKV (BR-PE243/2015) was used for the infection assays. After *in vitro* virus expansion, the concentration of the viral stock was determined through classical plaque assay and expressed as particle forming units (PFU)/mL. Adult IFNαβR^-/-^ mice previously immunized with ZK_ΔSTP or PBS control were infected two weeks after boost intravenously (iv) with 10^5^ particle forming units (PFU)/mL of ZIKV (100 μL). Survival and weight gain were monitored for up to 3 weeks. Sera were collected at 6- or 7-days post infection (dpi), while brains were obtained after euthanasia at 10 or 21 dpi and processed for viral load measurement. Mice were initially maintained until 21 dpi to observe clinical signs of infection, weight gain and mortality rates and possible natural recovery. The clinical signs of infection included observed neurological symptoms (hind limb paralysis, tremors and ataxia) and behavioral changes (changes in activity levels, exploration and social interactions). It is important to note that a non-lethal dose of virus was adopted to perform the infection challenge experiments. Therefore, no severe clinical signs of disease were observed to justify euthanasia. Mice that succumbed to infection presented weight loss, weakness and decreased social interaction. When immunization was performed in the presence of the Alum adjuvant formulation, the time point was reduced in half to confirm the vaccine’s protective and prophylactic effect.

### 2.7 Frequency of IFN-γ secreting cells by ELISPOT

Cellular response was assessed in spleens through ELISPOT using the IFN-γ ELISPOT set (BD Biosciences, USA) as previously described (26) 0.5x10^6^ cells/well were stimulated with 2 µg/mL of recombinant ZIKV E protein (Native Antigen, UK). The anti-CD3 and anti-CD28 antibodies (1 µg/mL each) (BD Bioscience, USA) were used as positive control. Unstimulated cells were used as baseline. Spots were quantified using AID *i*Spot FluoroSpot Reader System and AID EliSpot Version 7.0 software (AID-Autoimmun Diagnostika GMBH, Germany). Assays were performed in duplicate, baseline values were subtracted from all samples, and the results were expressed as the average number of spots forming cells (SFCs) per 10^6^ cells.

### 2.8 Titration of anti-ZIKV IgG antibodies by ELISA

To measure equilibrium binding affinity, anti-ZIKV E IgG antibodies were titrated through ELISA. High binding 96-well polystyrene plates (Costar, USA) were coated with 2 µg/mL of recombinant ZIKV E protein (Native Antigen, UK) for 16h at 4°C, and blocked with assay buffer (5% skimmed milk in PBS-T buffer) for 30 min at RT. Samples were serially diluted (1:50 – 1:25.600) and added to the plates. Plates were washed five times with PBS-T followed by peroxidase-conjugated anti-mouse IgG antibody (Sigma Aldrich, USA) for 1h at RT. After a final wash step, the reaction was revealed with TMB (KPL SureBlue ReserveTM) substrate for 30min at RT, stopped with 1N HCl and read at 450nm (OD 450nm) using a microplate spectrophotometer (Benchmark Plus, Bio-rad, USA). All samples were tested in duplicate, and the results were considered valid when intra-assay variability was below 20%. Antibody titers are expressed as EC_50_ and were calculated using a three-parameter nonlinear model. The data was plotted using GraphPad Prism version 7.0.

### 2.9 Titration of anti-ZIKV neutralizing antibodies by plate reduction neutralization test

ZIKV-specific neutralizing antibodies were assessed by PRNT following a modified protocol described in detail elsewhere (35). Mice sera were heat-inactivated (56°C for 30 min), serially diluted (1:50 – 1:25.600) in culture media (DMEM, GIBCO, USA) and incubated with 50 PFU of locally isolated ZIKV for 1h at 37°C under gentle agitation. The antibodies-virus complexes were transferred to a monolayer of Vero cells, and DMEM containing 1% (w/v) carboxymethyl cellulose was overlaid. The cells were incubated at 37°C, 5% CO_2_ for 7 days. Plaque number was calculated after fixation of the cells with 10% formaldehyde and staining with 0.2% crystal violet. The cut-off for PRNT positivity was defined based on a 50% reduction in plaque counts (PRNT50) compared to the positive control (ZIKV infected cells). ZIKV-specific neutralizing antibody titers were estimated using a four-parameter non-linear regression. Samples were considered positive when neutralizing antibody levels were ≥1:100.

### 2.10 Frequency of germinal center B cells by flow cytometry

The frequency of GC B cells from iLNs was assessed one week after boost as previously described (26). 2x10^6^ cells were stained with anti-CD3 (PerCP-Cy5.5 – Clone: 17A2), anti-B220 (APC – Clone: RA3-6B2), anti-CD95 (PE-Cy7 – Clone: Jo2) and anti-GL-7 (FITC – Clone: GL-7) antibodies (all from BD Biosciences, USA). Viability was determined using live/dead (Texas Red, Invitrogen, USA). A total of 500.000 events were collected and analyzed by flow cytometry (LSR Fortessa, BD Biosciences, USA) using FACS-Diva (BD Bioscience, USA) and FlowJo 10.0.6 (BD Bioscience, USA) software. Fluorescence minus one (FMO) controls were included to confirm proper compensation and to define positive signals.

### 2.11 Viral load quantification by qRT-PCR

Viral load quantification was performed in sera and brain tissues of ZIKV-infected mice through qRT-PCR/Taqman following a modified protocol described in detail elsewhere (36). Briefly, viral RNA was extracted from serum and brain tissues using the QIAamp Viral RNA kit (Qiagen, Germany) and the RNeasy Mini Kit, (Qiagen, Germany), respectively, according to the manufacturer’s instructions. ZIKV RNA was amplified using the TaqMan Fast Virus 1–8 Step Master Mix reagent (Applied Biosystems, USA) in a 7500 Fast Dx Real-Time PCR Instrument (Applied Biosystems, USA). The following primers and probes were used to quantify viral RNA: 5′-CCGCTGCCCACACAAG-3′ (forward primer); 5′-CCACTAACGTTCTTTTGCAGACAT-3′ (reverse primer), and FAM-AGCCTACCTTGACAAGCAGTCAGACACTCAA-MGB (probe). Infective viral particle amount in the samples was estimated after interpolation to a ZIKV standard curve run in each plate. The standard curve used in the PCR assays consisted of the same viral inoculum used to infect the animals (in the challenge experiments) and ranged from 10^6^ to 10^2^ PFU/mL. The virus concentration corresponding to the last point of the dilution curve (10^2^ PFU/mL) was considered as the assay limit of detection (dashed line). Positive and negative controls corresponded to purified viruses (from cell culture) and water, respectively.

### 2.12 Statistical analysis

Data were analyzed using the GraphPad Prism software (version 7.0). Comparison between groups was performed using non-parametric statistical tests. P values were considered statistically significant when p≤ 0.05. * *Mann-Whitney;* # *Kruskal-Wallis;* § *Multiple-t-test.*

## 3 Results

### 3.1 Construction of an optimized DNA vaccine against ZIKV and *in vitro* antigen expression

**Figure 1:**
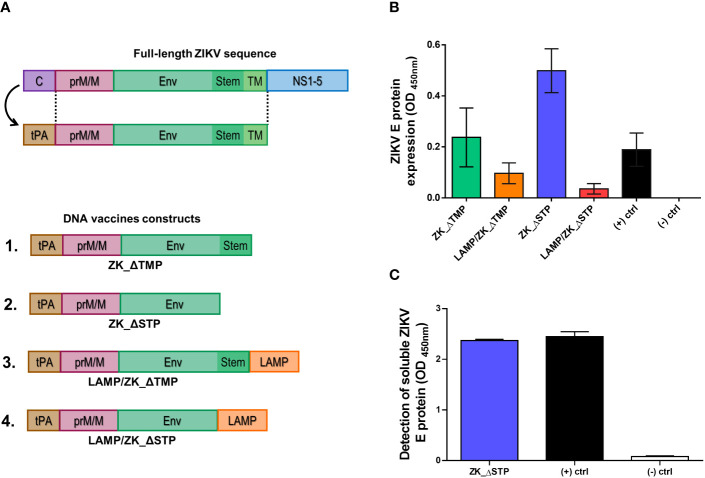
DNA vaccines design and antigen expression. **(A)** Schematic representation of the ZIKV genome and the ZIKV DNA vaccine constructs coding for the tPA-SP leader sequence and the ZIKV prM/M and E proteins. Constructs including a deletion of the TM (ZK_ΔTMP) and stem (ZK_ΔSTP) regions, as well as associated to LAMP (LAMP/ZK_ΔTMP and LAMP/ZK_ΔSTP), were also designed. **(B)** Detection of ZIKV E protein in the cytoplasm of BHK21 cells transfected with all four DNA vaccine constructs by cell-based ELISA. Empty vector (pcDNA3.1) and pL/YFV DNA vaccine were used as negative and positive controls, respectively. **(C)** Detection of soluble ZIKV E protein in the supernatant of BHK21 cells transfected with ZK_ΔSTP. Empty vector and purified ZIKV E protein were used as negative and positive controls, respectively.

Previous studies have demonstrated that prM/M and E proteins co-expression induces nAb against ZIKV (12, 14, 37) and others flaviviruses (38–40). Removal of antigen membrane anchors of E protein, such as the TM portion, often improves vaccine immunogenicity by favoring extracellular protein secretion (18, 19). Similarly, depletion of the E protein stem portion was also described to improve protective nAb production (13, 41, 42). We have previously demonstrated that humoral and cellular responses against viral antigens can be remarkably improved by the modulation of molecular adjuvants added to the expression cassette due to improved antigen presentation (23–26, 43). In this work, we designed four DNA vaccines coding for the ZIKV prM/M and E proteins along with the tPA-SP leader sequence (**Figure 1A**). The relevance of deletions of the TM (ΔTMP) and stem (ΔSTP) regions of envelope, as well as the presence of the LAMP molecular adjuvant, for vaccine immunogenicity were evaluated.

Aiming to define the best construct candidate for the *in vivo* studies, the vaccine antigens expression efficiency of all four constructs was assessed *in vitro*. While the ZIKV E protein was successfully detected intracellularly in transfected cells for all candidates (**Supplementary Figure 1**), the ZK_ΔSTP construct remarkably induced the highest antigen expression (**Figure 1B**). Additionally, the cell-secreted soluble E protein was also detected in the supernatants of cells transfected with ZK_ΔSTP (**Figure 1C**).

### 3.2 The magnitude of the immune response induced by the DNA vaccine constructs

Aiming to identify the most promising vaccine candidate, we assessed the immunogenicity of each construct in adult C57BL/6 mice. Mice were immunized via intradermal (id) route with two doses of each DNA vaccine at a 20-days interval, and humoral and cellular responses were interrogated one week after boost. Our research group has been largely using id administration in maternal and neonatal immunization protocols, especially due to easiness of access and vaccine bioavailability in neonatal mice (23–26).

**Figure 2:**
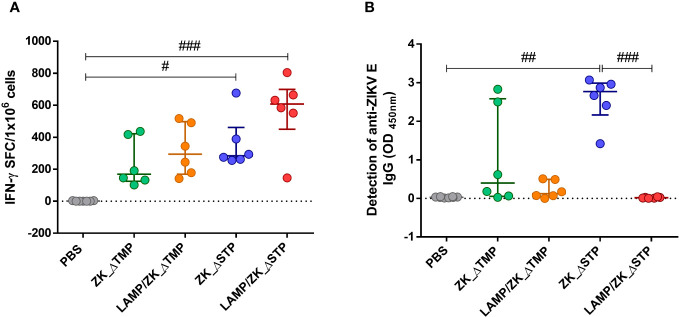
Magnitude of the ZIKV-specific cellular and humoral response upon immunization. Adult C57BL/6 WT mice were immunized (id) with two doses (50 μg in 50 μL of PBS) of ZK_ΔTMP, LAMP/ZK_ΔTMP, ZK_ΔSTP, LAMP/ZK_ΔSTP or PBS (negative control group), within 20-days interval and analysis performed one week after boost. **(A)** ZIKV-specific cellular response was evaluated in splenocytes by IFN-γ ELISPOT assay. **(B)** Detection of anti-ZIKV E IgG antibodies by ELISA in the sera. All measurements were performed in duplicate, and the results are expressed as median with interquartile range. *Kruskal-Wallis*: ^#^p≤0,05; ^##^p≤0,01; ^###^p≤0,001.

Vaccination with all four DNA constructs induced ZIKV-specific T cell response, but only vaccines without stem increased the frequency of IFN-γ-secreting cells in comparison to the PBS control group (**Figure 2A**). Mice immunization with ZK_ΔSTP vaccines showed higher levels of anti-ZIKV E IgG antibodies (**Figure 2B**), which suggests that deletion of the full membrane-anchoring regions of the E protein is fundamental to humoral response induction. The ZK_ΔSTP vaccine induced the strongest production of anti-ZIKV antibodies among the tested vaccines in comparison to the control group and LAMP/ZK_ΔSTP (**Figure 2B**). High levels of anti-ZIKV IgG2c antibody subclass were also detected upon ZK_ΔSTP immunization (**Supplementary Figure 2**). Noteworthy, vaccine formulations associated with LAMP did not show enhanced immunogenicity, when compared to their counterparts.

### 3.3 Immunogenicity of the ZK_ΔSTP DNA vaccine over time

**Figure 3:**
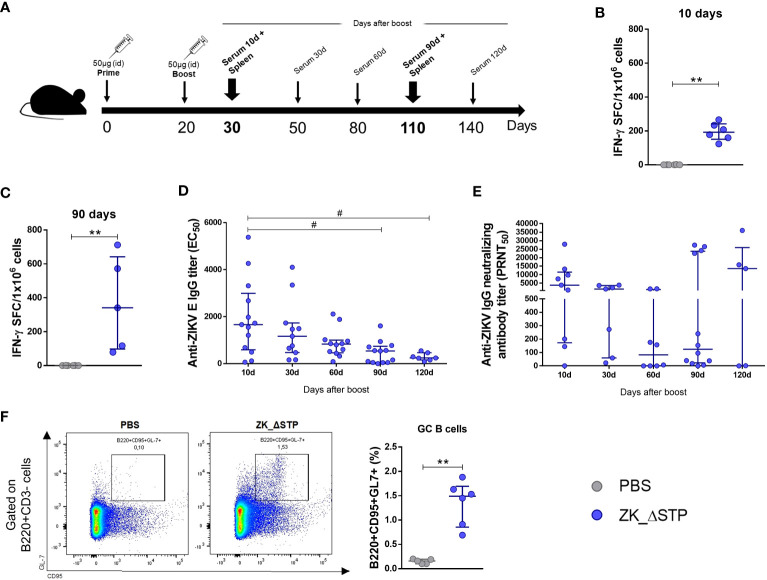
Assessment of short and long-term cellular and humoral response induced by the ZK_ΔSTP DNA vaccine over time. **(A)** Adult C57BL/6 WT mice were immunized (id) with two doses (50 μg in 50 μL of PBS) of ZK_ΔSTP or PBS (negative control group), within 20 days interval. **(B, C)** ZIKV-specific cellular response was evaluated in splenocytes obtained at 10 and 90 days after boost. **(D)** Measurement of equilibrium binding affinity of anti-ZIKV E IgG antibodies by ELISA in the sera. Antibody titers are expressed as EC_50_ and were calculated using a three-parameter nonlinear model. **(E)** Monolayers of Vero cells were cultivated during 7 days with 50 PFU of ZIKV pre-incubated with sera from immunized mice and neutralizing effect was expressed as PRNT50. **(F)** The frequency of GC B-cells (B220+CD95+GL-7+) was evaluated 7 days after the boost in iLNs by flow cytometry. All measurements were performed in duplicate, and the results are expressed as median with interquartile range. *Kruskal-Wallis*: ^#^p≤0,05. *Mann-Whitney*: **p≤0,01.

Based on the superior performance of the ZK_ΔSTP regarding antigen expression *in vitro* and induction of virus-specific immune response *in vivo*, this construct was selected for further validation. Mice were immunized as described before and immunogenicity of the selected vaccine was evaluated at different time points (**Figure 3A**). Our data on the frequency of IFN-γ-secreting cells shows that ZK_ΔSTP induced T cell response both in the short (10 days after boost) and the long-term (3 months after boost) assessment (**Figures 3B, C**)

As for B cell response, our data show that ZK_ΔSTP induced high titers of anti-ZIKV E IgG antibodies in the first month after immunization. However, these levels sharply decreased from 90 days on after immunization (**Figure 3D**). While this finding could indicate the loss of the long-lasting protective response, high nAb titers were still observed throughout the evaluation time (**Figure 3E**). This data was corroborated by the induction of GC B cells in the immunized animals (**Figure 3F**, **Supplementary Figure 3**). GC are transiently formed microanatomical sites within the B cell zone in the follicles of secondary lymphoid organs, where B cells proliferate and where antibodies undergo iterative rounds of gene somatic hypermutation coupled to multiple selection and differentiation pathways of affinity maturation. Therefore, GC B cell are known as the source of the high-affinity, class-switched antibodies required for protective immunity (44, 45). The increase in the frequency of GC B cells in draining iLN one week after the vaccination boost suggest that ZK_ΔSTP activated this cell population, which might be involved in efficient nAb generation.

### 3.4 Protective effect of the ZK_ΔSTP DNA vaccine against ZIKV challenge

**Figure 4:**
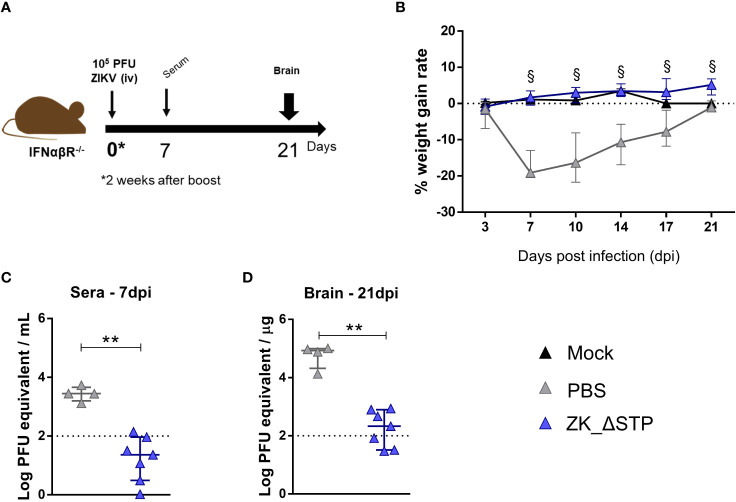
Protection of adult mice against ZIKV challenge upon ZK_ΔSTP vaccination. **(A)** Two weeks after the vaccine boost, adult C57BL/6 IFNαβR^-/-^ mice immunized with ZK_ΔSTP or PBS were infected with 10^5^PFU of ZIKV (iv) and evaluated for up to 3 weeks. **(B)** Weight gain was monitored for 21 days post infection (dpi). **(C, D)** ZIKV viral load was measured by qRT-PCR in sera obtained at 7dpi and brain tissues at 21dpi. All measurements were performed in duplicate, and the results are expressed as median with interquartile range. The dashed line represents the detection limit of the assay. *Multiple t test*: ^§^p≤0,05. *Mann-Whitney*: **p≤0,01.

Adult C57BL/6 IFNαβR-/- mice, known as the susceptible model of ZIKV infection, were used for the viral challenge assays. Prior to the challenge experiments, ZK_ΔSTP vaccine immunogenicity was confirmed in this mice lineage. Production of virus-specific and neutralizing antibodies were identified in sera obtained at day zero before infection (**Supplementary Figures 4A, B**). To assess vaccine protective efficacy, mice were challenged with 10^5^ PFU of ZIKV two weeks after the ZK_ΔSTP vaccine boost and evaluated regarding weight gain, viremia and brain viral load for up to 3 weeks (**Figure 4A**).

The data showed that one mouse from non-immunized (PBS) group sustained lethality (20%), while survival was maintained in mice vaccinated with ZK_ΔSTP. PBS group mice also exhibited marked weight loss in the first week after infection and presented a slower recovery, when compared to animals immunized with the ZK_ΔSTP vaccine (**Figure 4B**). Noteworthy, the ZK_ΔSTP immunized mice exhibited a weight gain profile comparable to the non-infected group (mock) and showed no clinical signs of infection. Additionally, viremia was absent in the vaccinated mice 1 week after infection (**Figure 4C**). Protective effect of vaccination was also prominent in the brain tissues (**Figure 4D**). We observed a considerable decrease in viral load in the brains at 21dpi (samples from 42% of animals were under the detection limit), when compared to the PBS group. Interestingly, the presence of ZIKV in the brain tissues of PBS group was observed even 3 weeks post infection (**Figure 4D**). Despite the promising protective effect observed, the lack of a sterile immunity indicates that additional strategies might be needed to increase antibody titers over time and to ensure optimal immunity.

### 3.5 Association of adjuvants to the ZK_ΔSTP immunization as a strategy to boost vaccine response and its protective effect

**Figure 5:**
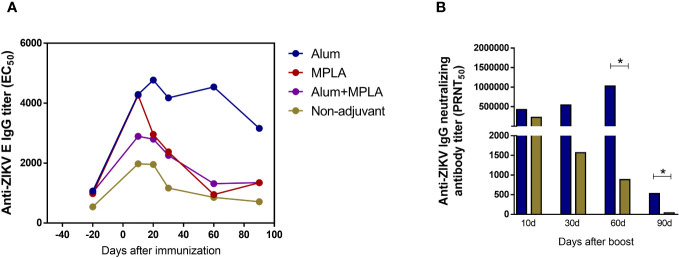
Magnitude of the humoral response induced by immunization with ZK_ΔSTP combined with commercial adjuvants. Adult C57BL/6 mice were immunized with two doses of 50µg (id) of the ZK_ΔSTP DNA vaccine alone (non-adjuvant) or in the presence of Alum (10 mg/mouse), MPLA (10 μg/mouse) or Alum+MPLA adjuvants. **(A)** Titration of anti-ZIKV E IgG antibodies in sera by ELISA. **(B)** Titration of anti-ZIKV neutralizing antibodies by PRNT in the sera from mice immunized with ZK_ΔSTP DNA vaccine only or in association with Alum. All measurements were performed in duplicate, and the results are expressed as median. *Mann-Whitney*: *p≤0,05.

Aiming to enhance the magnitude and lasting of the vaccine humoral response, immunization of the ZK_ΔSTP DNA vaccine along with commercially available adjuvants was explored. Animals were immunized following the same protocol previously described and in association with Alum, MPLA or a combination of both (Alum+MPLA). Immunization with MPLA alone and its combination with Alum (Alum+MPLA) did not increase virus-specific antibody titers. In fact, those groups exhibited a similar IgG profile to what was observed for the non-adjuvant control group (animals that received the ZK_ΔSTP DNA vaccine only), in which antibody levels decreased 2 months after boost (**Figure 5A**). Conversely, immunization in the presence of Alum induced high titers of anti-ZIKV E IgG antibodies over time (**Figure 5A**), as well as increased anti-ZIKV nAb titers at short and long-term periods, when compared to the non-adjuvant group (**Figure 5B**). Interesting to mention that, even though Alum induced a neutralizing response similar to the control group during the first month after immunization, long-term nAb levels were significantly increased later on (60 and 90 days after boost). This finding reflects an important improvement of vaccine humoral response induction (**Figure 5B**). Based on these results, the immunization with ZK_ΔSTP in association with Alum was further evaluated for protection against ZIKV infection.

**Figure 6:**
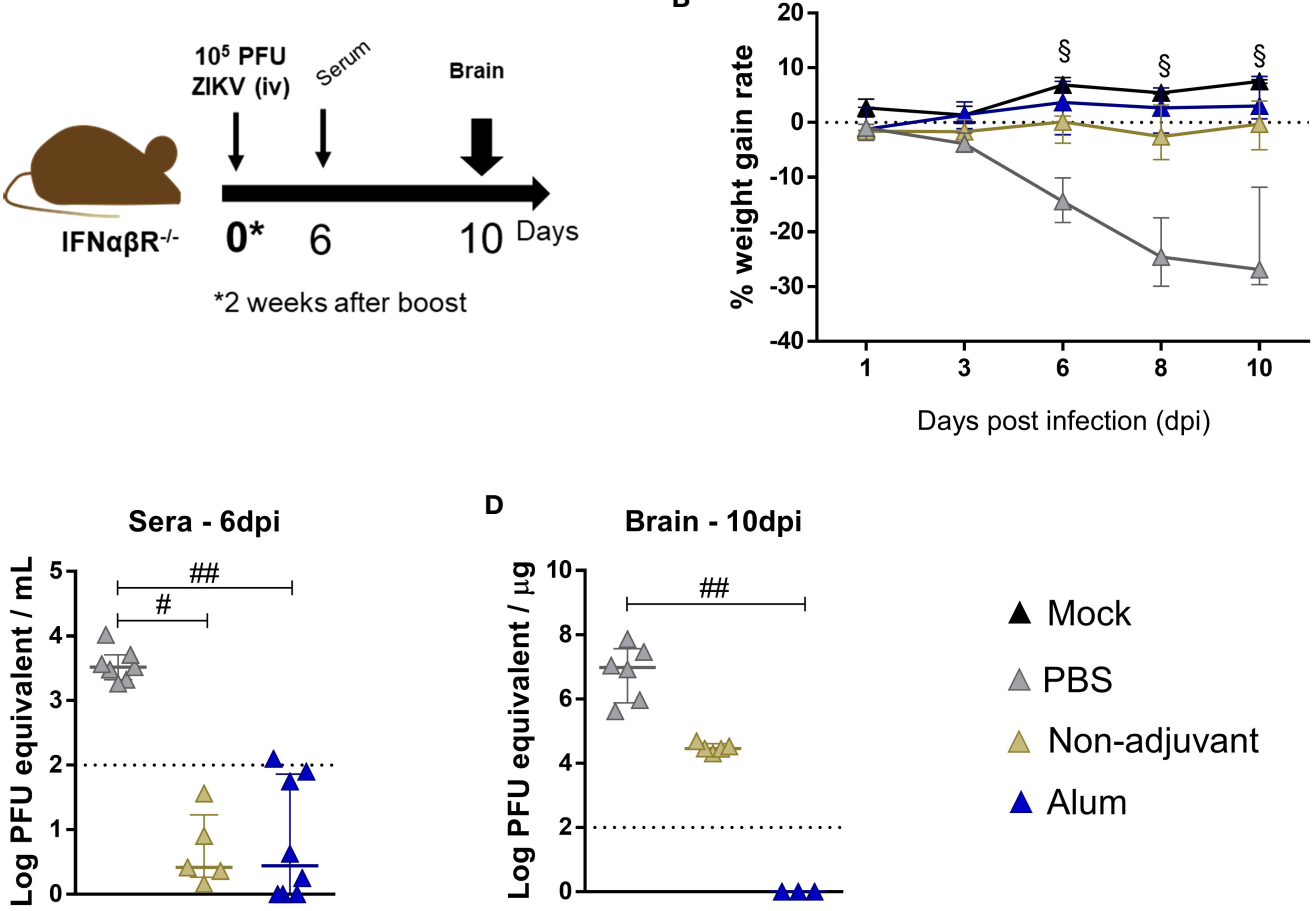
Protection of adult mice against ZIKV challenge upon ZK_ΔSTP+Alum vaccination. **(A)** Two weeks after the vaccine boost, adult C57BL/6 IFNαβR^-/-^ mice immunized with ZK_ΔSTP (non-adjuvant) or ZK_ΔSTP+Alum were infected with 10^5^ PFU of ZIKV (iv) **(B)** Weight gain was evaluated for up to 10dpi. ZIKV viral load was measured by qRT-PCR in sera **(C)** and brain **(D)** obtained at 6 and 10dpi, respectively. All measurements were performed in duplicate, and the results are expressed as median with interquartile range. The dashed line represents the detection limit of the assay. *Multiple t test*: ^§^p≤0,05. *Kruskal-Wallis*: ^#^p≤0,05; ^##^p≤0,01.

Adult IFNα/βR-/- mice were challenged for ZIKV infection following the same protocol previously adopted (**Figure 6A**), and sera and brains were collected at 6 and 10dpi, respectively. As previously observed, 2 animals sustained lethality (25%) in the non-vaccinated group (PBS), while no animal loss was observed in the immunized groups. Additionally, weight gain rate was maintained within normal limits for these groups (when compared to the mock control) (**Figure 6B**). It is important to mention that, while immunization with ZK_ΔSTP alone and its combination with Alum lead to elimination of viremia within one week after infection (**Figure 6C**), only the immunization in the presence of the adjuvant was capable of ensuring complete absence of virus in the brain at short period (**Figure 6D**). Together, these findings confirm the protective effect of this vaccine approach.

## 4 Discussion

High morbidity associated to congenital ZIKV infection still remains as a relevant public health problem and highlights the pressing need for the development of prophylactic vaccines against this infection. The vaccines against ZIKV currently under study often aim to induce strong neutralizing humoral response, as this is well known as the major mechanism and correlate of protection (12, 46). In this work, we have developed a DNA vaccine and optimized its performance by modulating the length of the target viral antigens and by associating this vaccine to adjuvants aiming to achieve strong protective efficacy.

Among our vaccine designs, the ZK_ΔSTP vaccine candidate was identified as the most capable of inducing effective long-term cellular response and the generation of nAb (**Figures 3C, E**). Our data indicate that removal of the TM portion only is not enough to boost the expression of vaccine-encoded viral proteins and to trigger a protective immune response, which is in agreement with others studies on the development of Zika vaccines and Zika-based vaccines (13, 19, 41, 42, 47). Instead, the depletion of the complete membrane-anchoring region (stem and TM) seems to be pivotal to stimulate APCs and B cells, and to lead to proper immunological response (**Figures 2**, **3**). This is possibly due to improved antigen presentation and enhanced vaccine proteins expression and stability. As there is no consensus on the precise length of the stem region within the E protein (48, 49), we removed the largest range of stem coverage based on the current literature.

Based on previous studies on the use of molecular adjuvants to boost vaccine efficacy in animal models, a sequence coding for the LAMP molecule and the tPA-SP leader sequence were evaluated. In contrast to other studies where LAMP enhanced vaccine efficacy (18, 24, 26, 43, 50), in our vaccine model, association of the LAMP molecule to the target viral proteins did not induce strong protein expression *in vitro* or strong humoral response *in vivo* (**Figure 1B** and **Figure 2B**). Additionally, the combined use of LAMP and tPA-SP on the same constructs did not provide improved vaccine immunogenicity either. We hypothesized that this is likely due to the distinct roles of those molecules in antigen presentation: while LAMP leads to antigen processing and presentation on the cell surface through MHC-II molecules, tPA-SP leads to antigen secretion. This hypothesis is supported by the fact that there are no studies describing the combined use of LAMP and tPA-SP for vaccine development, to the best of our knowledge.

Additionally, our data show that mounting an effective neutralizing response against ZIKV seems to require proper B cell stimulation by recognition of well-folded viral proteins. The presence of tPA-SP leader sequence seems to enhance immunogenicity as shown in our best vaccine candidate (ZK_ΔSTP) by potentializing the secretion of folded viral antigens (**Figure 1C**). This is in agreement with other studies showing that flavivirus breadth during the cell infection process leads to the exposition of cryptic epitopes, but with different degrees of accessibility to neutralizing antibodies (51–53). One of these epitopes is the linear and highly conserved fusion loop in the envelope domain II (in the E dimer interface) (41, 54). Antibodies targeting this epitope might cause steric hindrance and inhibit the insertion of the E protein into the endosomal membrane, therefore blocking the cell infection process (41, 55). Corroborating the hypothesis of proper B-cell stimulation, the ZK_ΔSTP vaccine also induced the generation of GC B cells (**Figure 3F**). Formation of GC sites is crucial to ensure effective humoral response, production of long-lived antibody secreting plasma cells and memory B cells (56). Additionally, the induction of GC response has been associated to improved immunogenicity upon administration of genetic vaccines (26, 57).

It is important to mention that cellular composition of the skin (including the presence of different subsets of APCs differentially distributed across different regions of the skin), variable vascularity, dose and volume sensitivity (possibly leading to variability in the vaccine dispersion within the skin layer) have been described to influence the magnitude of the immune response in mice (58). This could lead to heterogeneity in the response within mice groups, with a similar profile as it has been observed in previous studies using DNA vaccination (26). On the other hand, several published works have also shown that intradermal immunization can lead to strong immune activation following vaccination with different technologies in mice and humans and using low vaccine doses (59–63).

Here the immunizations were performed without any other delivery method since the primary aim in this study was to assess the influence of ZIKV antigens length modulation on the vaccine immunogenicity. Even though previous genetic vaccines studies have emphasized the need of the full-length ZIKV E protein or the depletion of the TM portion only to achieve satisfactory immunogenicity, those vaccines were delivered through different methods [*e.g.*, electroporation, lipid nanoparticles or adenovirus vectors (14, 15, 19, 64, 65)], which themselves are expected to increase immunogenicity.

Immunization with the ZK_ΔSTP DNA vaccine showed protective effect at short-term upon viral challenge (**Figures 4B–D**). Immunized mice showed no clinical signs of ZIKV infection and exhibited significant decrease of viral load in brain tissues after 3 weeks after infection (**Figures 4B, D**). Despite of this, the antibody response was not sustained over time and showed a sharp decline in virus-specific IgG antibodies titers after 60 days after boost (**Figure 3D**). This finding indicates a decline in long-term protection and is likely the reason why we did not observe sterile protection in our assays.

The association of the ZK_ΔSTP vaccine with the Alum adjuvant was able to overcome this limitation and to favor the development of a long-term neutralizing humoral response (**Figure 5B**). This was somewhat expected, given the well-documented success of Alum in several licensed vaccine formulations (66). The magnitude of the humoral response was markedly improved, which lead to enhanced vaccine protective efficacy. Animals immunized with ZK_ΔSTP+Alum were protected from ZIKV challenge, which can be noted by the absence of virus RNA in the brain tissues at 10dpi (**Figure 6D**). Noteworthy to mention that protection was achieved twice as early as what we had observed in previous experiments when animals received the ZK_ΔSTP vaccine only, and viral RNA was quantified at 21dpi (**Figure 4D**). Long-term neutralizing response and congenital protection still need to be addressed in future studies.

In conclusion, we have developed a DNA vaccine against ZIKV that protect adult mice from getting infected. We showed that removal of the ZIKV E membrane-anchoring regions and the secretion of viral antigens are fundamental to lead to proper immune response activation in animal model. We also showed that association of our vaccine to Alum adjuvant is required to ensure sustained long-term protective response. Our findings advance understanding of the rational development of vaccine strategies for ZIKV and other flaviviruses, as well as highlight key factors that might be fundamental to the future development of vaccines to protect women of childbearing age against ZIKV infection.

## Data availability statement

The raw data supporting the conclusions of this article will be made available by the authors, without undue reservation.

## Ethics statement

The animal study was approved by Ethics Committee of Animal Care and Use (CEUA-ICB: 6912040918, CEUA-IMT: 363 and CEUA-FMUSP: 1763/2022). The study was conducted in accordance with the local legislation and institutional requirements.

## Author contributions

FT: Conceptualization, Investigation, Methodology, Writing – original draft, Funding acquisition. LO: Investigation, Methodology, Writing – original draft. AB: Investigation, Methodology, Writing – original draft. RA: Investigation, Methodology, Writing – original draft. ES: Investigation, Methodology, Writing – original draft. BL: Investigation, Methodology, Writing – original draft. WA: Investigation, Methodology, Writing – original draft. AD: Resources, Writing – original draft. RL: Conceptualization, Resources, Writing – original draft, Funding acquisition. MS: Conceptualization, Resources, Supervision, Writing – original draft, Funding acquisition, Writing – review & editing. IV: Conceptualization, Investigation, Methodology, Supervision, Writing – original draft, Writing – review & editing, Funding acquisition.
