# Corrigendum: Enhanced immunogenicity and protective efficacy in mice following a Zika DNA vaccine designed by modulation of membrane-anchoring regions and its association to adjuvants

**DOI:** 10.3389/fimmu.2024.1376059

**Published:** 2024-02-01

**Authors:** Franciane Mouradian Emidio Teixeira, Luana de Mendonça Oliveira, Anna Cláudia Calvielli Castelo Branco, Ricardo Wesley Alberca, Emanuella Sarmento Alho de Sousa, Bruno Henrique de Sousa Leite, Wenny Camilla dos Santos Adan, Alberto José da Silva Duarte, Roberto Dias Lins, Maria Notomi Sato, Isabelle Freire Tabosa Viana

**Affiliations:** ^1^ Laboratory of Dermatology and Immunodeficiencies, LIM-56, Department of Dermatology, Tropical Medicine Institute of São Paulo, University of São Paulo Medical School, São Paulo, Brazil; ^2^ Department of Immunology, Institute of Biomedical Sciences, University of São Paulo, São Paulo, Brazil; ^3^ Department of Virology, Aggeu Magalhães Institute, Oswaldo Cruz Foundation, Recife, Brazil

**Keywords:** DNA vaccine, Zika virus, envelope protein, membrane-anchoring regions, adjuvants, protection, immunogenicity

In the published article, there was an error regarding the author list for Maria Notomi Sato and Isabelle Freire Tabosa Viana. As well as having being the corresponding author, they should also have *“These authors share senior authorship.”*.

The authors apologize for this error and state that this does not change the scientific conclusions of the article in any way. The original article has been updated.

