# Supplementary material for: Enhanced immunogenicity and protective efficacy in mice following a Zika DNA vaccine designed by modulation of membrane-anchoring regions and its association to adjuvants
Source: Front Immunol. 2024 Jan 19;15:1307546. doi: 10.3389/fimmu.2024.1307546 (PMC10867427; doi:10.3389/fimmu.2024.1307546)
Supplement: Supplementary file 1 [file DataSheet_1.docx]

***Supplementary Material***

**Enhanced immunogenicity and protective efficacy in mice following a Zika DNA vaccine designed by modulation of membrane-anchoring regions and its association to adjuvants**

1. **Supplementary methods**
   1. **Intracellular identification of ZIKV envelope protein by immunofluorescence**

Monolayers of BHK-21 cells (ATCC, USA) were seeded over coverslips in 6 well plates a day in advance. Cells were transfected with the four DNA constructs using Lipofectamine 2000 (Invitrogen, USA) according to the manufacturer’s instructions. A plasmid coding for YFV proteins (pL/YFV) was used as a transfection positive control. After 48h, the culture supernatant was removed, and the cells were fixed with 1 mL of paraformaldehyde (PFA, 4%, Sigma Aldrich, USA) for 1 hour, and permeabilized with 300 μL of Triton X-100 (0.1%, 15 min). Cells were washed 3 times with PBS and incubated for 15 minutes with 300 μL ammonium chloride (50mM), followed by 15 minutes of blocking buffer (PBS / 3% BSA, Sigma Aldrich, USA). For ZIKV E detection, the 4G2 monoclonal antibody (1:1000 diluted in PBS) was added for 2h at room temperature (RT). Subsequently, cells were stained with anti-mouse IgG FITC (1:500 diluted in PBS) for an additional hour. A new wash cycle was performed, and the slides were mounted using 7 μl of Prolong Gold Antifade + DAPI (Thermo, USA). Images were acquired using a Leica DMI8 fluorescence microscope (objective: 63X) and the fluorescence corresponding to the expression of the target protein (in green) was captured in the GFP channel.

- 1. **Detection of anti-ZIKV IgG2c antibody subclass by ELISA**

The detection of anti-ZIKV E IgG2c subclass antibodies was performed in mice sera obtained 10 days after boost. High binding, half area 96-well polystyrene plates (Costar, USA) were coated with 2 µg/mL of recombinant ZIKV E protein (Native Antigen, UK) for 16 h at 4 °C. The reactions were blocked with assay buffer 5% skimmed milk (Bio-Rad, USA) in PBS-T buffer (1X PBS with 0.05% Tween-20) for 30 min at RT. Samples (dilution 1:50) were added in assay buffer for 2h at room temperature. Plates were washed five times with PBS-T followed by peroxidase-conjugated anti-mouse IgG2c antibody (Sigma Aldrich, USA) for 1 h at RT. After a final wash step, the reaction was revealed with tetramethylbenzidine (TMB, KPL SureBlue Reserve, USA) substrate for 30 min at RT, stopped with 1 N HCl and read at 450 nm (OD 450nm) using a microplate spectrophotometer (Benchmark Plus, Bio-rad, USA). All samples were tested in duplicate, and the results were considered valid when intra-assay variability was below 20%.

1. **Supplementary Figures**
   1. **Supplementary Figure 1**

**
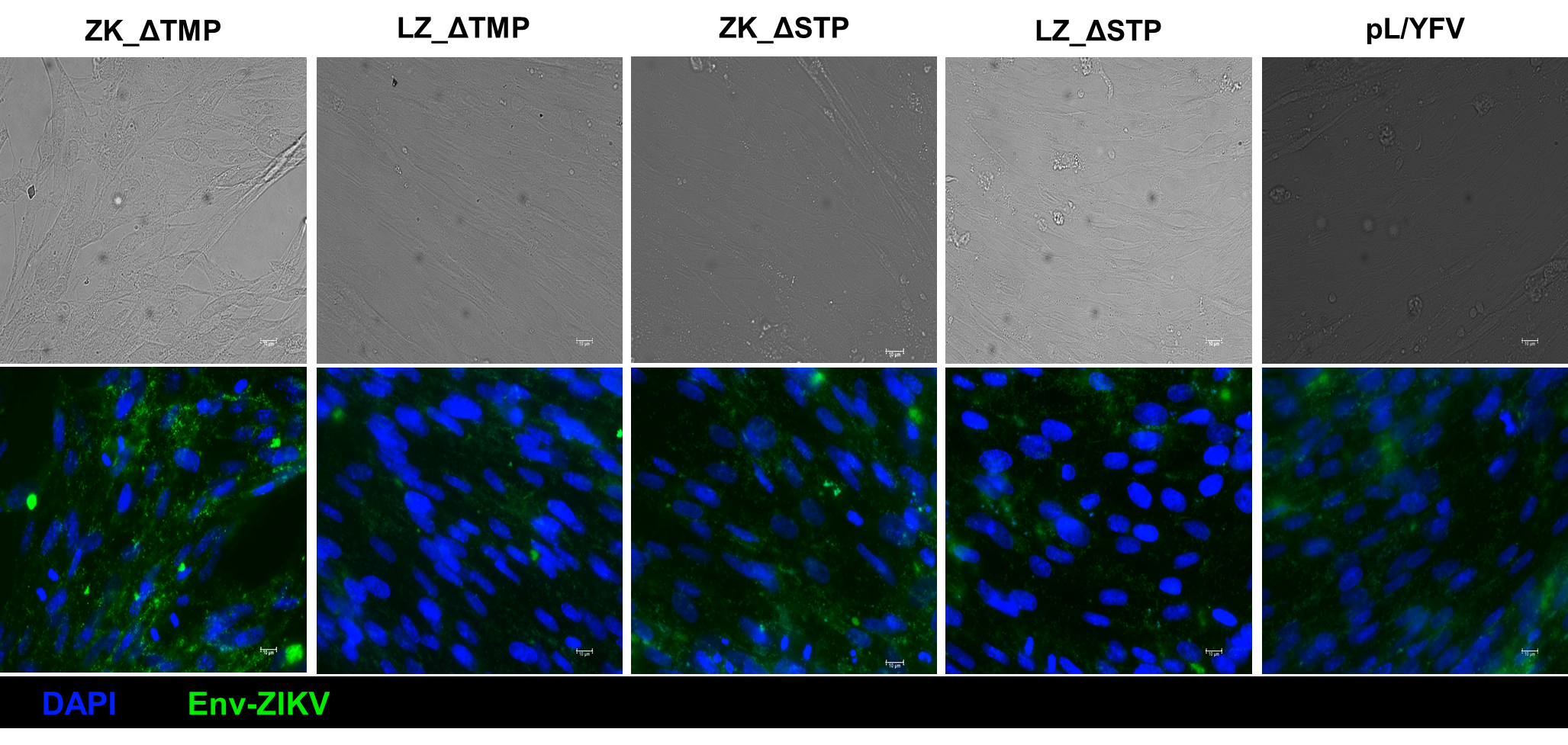
**

**Supplementary Figure 1.** ZIKV E protein expression *in vitro*. BHK21 cells were transfected with all four DNA vaccine constructs and the pL/YFV control plasmid. Envelope protein expression was identified by immunofluorescence (objective: 63X; scale bar: 10 μm) after 48 h post transfection.

- 1. **Supplementary Figure 2**

**Supplementary Figure 2.** Detection of ZIKV-specific IgG2c antibodies induced by ZK_ΔSTP immunization. Adult C57BL/6 WT mice were immunized (id) with two doses (50 μg in 50 μL of PBS) of ZK_ΔSTP. The anti-ZIKV E IgG2c antibody subclass was detected in sera obtained 10 days after boost by ELISA (at a 1:50 dilution). All measurements were performed in duplicate, and the results are expressed as median with interquartile range.

- 1. **Supplementary Figure 3**

**
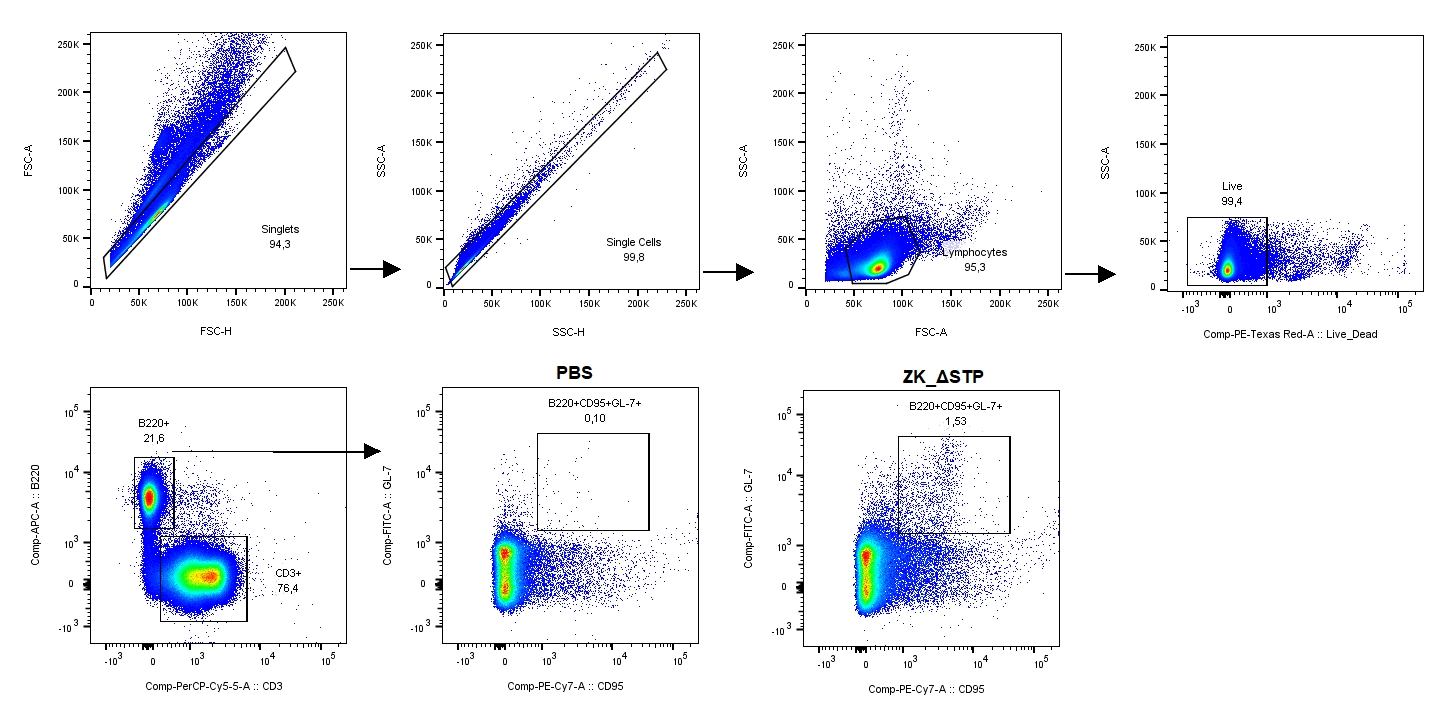
**

**Supplementary Figure 3.** Gate strategy to analyze GC B cells by flow cytometry. Adult C57BL/6 WT mice were immunized (id) with two doses (50 μg in 50 μL of PBS) of ZK_ΔSTP or PBS (negative control group), within 20 days interval. The frequency of GC B-cells was evaluated 7 days after the boost in iLNs by flow cytometry. GC B cells populations was defined within live cells according to phenotype B220+CD95+GL-7+.

- 1. **Supplementary Figure 4**


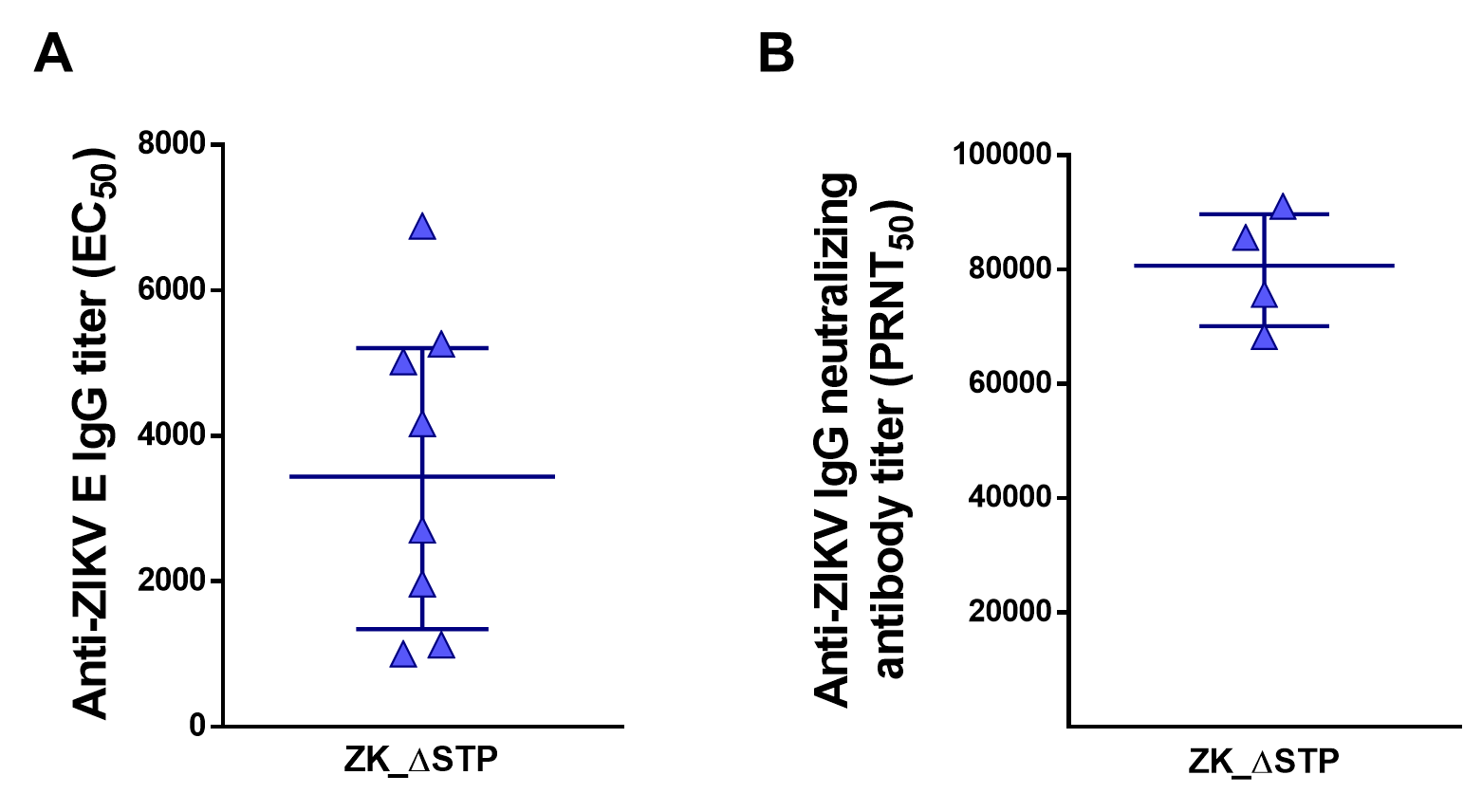


**Supplementary Figure 4.** Antibody response of ZK_ΔSTP DNA vaccine in C57BL/6 IFNαβR^-/-^ mice. Sera obtained from adult C57BL/6 IFNαβR^-/-^ mice immunized with ZK_ΔSTP two weeks after boost were evaluated for anti-ZIKV E IgG antibodies titers by ELISA **(A)** and neutralizing activity by PRNT **(B)**. Assays were performed in duplicate, and the results are expressed as median with interquartile range.
